# Ultra-high-resolution assessment of lesion extension after cryoballoon ablation for pulmonary vein isolation

**DOI:** 10.3389/fcvm.2022.985182

**Published:** 2022-11-09

**Authors:** Francesco Spera, Maria Lucia Narducci, Gianluigi Bencardino, Francesco Perna, Antonio Bisignani, Gaetano Pinnacchio, Claudio Tondo, Ruggero Maggio, Giuseppe Stabile, Saverio Iacopino, Fabrizio Tundo, Anna Ferraro, Antonio De Simone, Maurizio Malacrida, Federico Pintus, Filippo Crea, Gemma Pelargonio

**Affiliations:** ^1^Department of Cardiovascular Sciences, Fondazione Policlinico Universitario Agostino Gemelli Istituto di Ricovero e Cura a Carattere Scientifico (IRCCS), Rome, Italy; ^2^Centro Cardiologico Monzino IRCCS, Milan, Italy; ^3^Infermi Hospital, Rivoli, Italy; ^4^Laboratorio di Elettrofisiologia, Clinica San Michele, Maddaloni, Italy; ^5^Mediterranea Cardiocentro, Naples, Italy; ^6^Maria Cecilia Hospital, Ravenna, Italy; ^7^Boston Scientific, Milan, Italy; ^8^Institute of Cardiology, Catholic University of Sacred Heart, Rome, Italy

**Keywords:** atrial fibrillation, ablation < electrophysiology, cryoballoon ablation, scar ablation, electrograms

## Abstract

**Introduction:**

Unrecognized incomplete pulmonary vein (PV) isolation during the index procedure, can be a major cause of clinical recurrences of atrial fibrillation (AF) after cryoballoon (CB) ablation. We aimed to characterize the extension of the lesions produced by CB ablation and to assess the value of using an ultra-high resolution electroanatomic mapping (UHDM) system to detect incomplete CB lesions.

**Materials and methods:**

Twenty-nine consecutive patients from the CHARISMA registry undergoing AF ablation at four Italian centers were prospectively evaluated. The Rhythmia™ mapping system and the Orion™ (Boston Scientific) mapping catheter were used to systematically map the left atrium and PVs before and after cryoablation.

**Results:**

A total of 116 PVs were targeted and isolated. Quantitative assessment of the lesions revealed a significant reduction of the antral surface area of the PV, resulting in an ablated area of 5.7 ± 0.7 cm^2^ and 5.1 ± 0.8 cm^2^ for the left PV pair and right PV pair, respectively (*p* = 0.0068). The mean posterior wall (PW) area was 22.9 ± 2 cm^2^ and, following PV isolation, 44.8 ± 6% of the PW area was ablated. After CB ablation, complete isolation of each PV was documented by the POLARMap™ catheter in all patients. By contrast, confirmatory UHDM and the Lumipoint™ tool unveiled PV signals in 1 out of 114 of the PVs (0.9%). Over 30-day follow-up, no major procedure-related adverse events were reported. After a mean follow-up of 333 days, 89.7% of patients were free from arrhythmia recurrence.

**Conclusion:**

The lesion extension achieved by the new CB ablation system involved the PV antrum, with less than 50% of the PW remaining untouched. The new system, with short tip and circular mapping catheter, failed to achieve PV isolation in only 0.9% of all PVs treated.

**Clinical trial registration:**

[http://clinicaltrials.gov/], identifier [NCT03793998].

## Introduction

In paroxysmal atrial fibrillation (AF), pulmonary vein isolation (PVI) by means of a cryoballoon (CB) has been associated with better results than radiofrequency ablation, in terms of safety and long-term efficacy, and displays less inter-operator variability in terms of success (1, 2). Recently, a new CB technology (POLARx; Boston Scientific) for PVI in patients with AF has been introduced. This new CB ablation system, compared to previous system, includes a more compliant 28 mm balloon catheter, designed to maintain the same volume during application of cryoenergy. Moreover, a difference can be found in the gas pressure, that is kept stable and lower in POLARx system (2.5 psi), while it increases to higher values during cryoenergy applications with previous system (3–7).

Theoretically the semi-elastic thermoplastic material used for this balloon and the constant pressure during the ablation phase could allow for better compliance and stability during veins occlusion. After CB ablation by means of the standard technology, characterization of the lesion set has revealed a large area of antral PVI in the acute phase and significant regression of the cryoablated zone during second procedures (8, 9). Only one study, involving 8 patients, has evaluated acute lesion characteristics after PVI with the POLARx™; this showed a large antral lesion and ablation of 50% of the posterior wall (10). The use of high-resolution mapping during CB ablation is useful not only in order to characterize the tissue, but also to detect incomplete PVI unrecognized by means of conventional circular mapping catheter mapping (11–13). A new map analysis tool (Lumipoint™, Boston Scientific) automatically identifies fragmented potentials and continuous activation, and seems to detect residual antral potentials after PVI better than other tools (14).

The aim of this study was to describe the extent and morphology of PVI lesions, in order to determine the rate of unidentified incomplete PVI and to quantify residual antral potentials after CB ablation with the new POLARx™ system, which uses ultra-high-resolution mapping (UHDM).

## Materials and methods

### Patient population and study design

The Catheter Ablation of Arrhythmias with a High-Density Mapping System in the Real-World Practice (CHARISMA) (ClinicalTrials.gov Identifier: NCT03793998) is a prospective, single-arm, multicenter, continued-access cohort study designed to describe clinical practice regarding the approach to the ablation of various arrhythmias. The study complies with the Declaration of Helsinki, the locally appointed ethics committee approved the research protocol, and informed consent was obtained from all patients prior to enrollment.

Patients were enrolled in four Italian centers. The study population consisted of 29 consecutive patients with symptomatic paroxysmal AF who underwent PVI by means of a novel CB system. High-resolution 3D atrial mapping was performed before and immediately after PVI, in order to assess lesion characteristics. AF was defined according to the 2017 expert consensus statement (15). The patients enrolled had a history of failed rhythm control with class I or III antiarrhythmic drugs.

### Ablation procedure

Imaging procedures and the management of antiarrhythmic and anticoagulation medication up to the time of the procedure were at the discretion of the operator, in accordance with the standard workflow adopted at each institution.

The ablation procedure was performed under conscious sedation. A decapolar (Dynamic XT™, Boston Scientific, Marlborough, MA, USA) and a quadripolar catheter were placed in the coronary sinus and superior vena cava (SVC), respectively, to obtain a stable reference for the Rhythmia mapping system and for the detection of phrenic stimulation. After single transseptal puncture under fluoroscopic guidance or intracardiac echocardiography, intravenous unfractionated heparin boluses were administered, in order to maintain an activated clotting time of >300 s.

The basket mapping catheter (IntellaMap Orion, Boston Scientific, Marlborough, MA, USA) was introduced through the sheath and used in combination with the Rhythmia HDx mapping system to create a 3-dimensional UHDM of the left atrium in sinus rhythm.

After obtaining a bipolar voltage map of the left atrial substrate, we switched to the steerable 12.7Fr POLARSHEATH under fluoroscopy control. Once the sheath had been connected to a continuous saline flush, the short-tip (5 mm) POLARx CB (28 mm) was inserted into the left atrium over a circular inner lumen octopolar mapping catheter/guidewire (POLARMAP). The POLARMap was connected through the breakout box to the Rhythmia HDx mapping system, to allow POLARMap visualization in the acquired 3D map, which helped to cannulate the four veins without using fluoroscopy.

Prior to each application, PV occlusion was verified by means of contrast venography, and the CB was positioned until total contrast retention had been achieved without visible leaks during contrast injection.

The application time for each vein was calculated as the time-to-isolation (TTI), and the circular mapping catheter was positioned at the proximal part of the ostium before each freeze (16). A standard 180 s cryoenergy application was delivered if the TTI was 60 s or less; otherwise, the application was prolonged to 240 s. An additional CB application was performed in patients displaying a PV potential after the first application.

In order to avoid phrenic nerve palsy, continuous high-output pacing to capture the phrenic nerve was performed during right-PV applications. Cryoablation was immediately stopped if there was a loss or reduction of phrenic nerve capture. Specifically, a movement sensor (Diaphragm Movement Sensor, DMS, Boston Scientific), in addition to conventional methods, was used to check nerve capture during CB ablation.

Acute entry block (absence of local PV potential inside the vein) and paced exit block (using any pair of electrodes of the circular catheter) were verified at the end of the procedure by means of the POLARMap™, which was placed at the ostium of every PV. Pacing from the distal tip of the coronary sinus catheter or superior vena cava catheter was used to distinguish far-field atrial signals from PV potentials recorded on the circular mapping catheter.

### Mapping procedure

Ultra-high-resolution 3-D left atrium mapping was performed by means of the Rhythmia™ system in all patients before and immediately after PVI. A multipolar mini-basket catheter (IntellaNAV Orion™) consisting of 8 splines with a total of 64 electrodes, an inter-electrode spacing of 2.5 mm and surface area of each electrode of 0.4 mm^2^, was inserted through the POLARSHEATH™ to create a bipolar voltage map of the left atrium, before and after CB-PVI, in sinus rhythm. The left atrial shell was acquired in expiration timing, and the system was programmed with strict criteria of cycle length stability, coronary sinus activation and electrode positioning for automatic mapping. In one case, in which the PV was not isolated by means of the CB, a touch-up application with an irrigated radiofrequency ablation catheter (IntellaNAV™ Mifi OI, Boston Scientific Boston Scientific, Marlborough, MA, USA) was performed; this was guided by the local impedance drop, targeting a minimum local impedance drop of 10 Ω within 30 s, and was stopped when a maximum cutoff local impedance drop of ≥40 Ω was observed. After radiofrequency touch-up, a new map of the PV was acquired in order to confirm entry and exit block.

Electrograms with voltages above 0.5 mV were considered to indicate healthy and unablated tissue, while EGMs with voltages less than 0.2 mV were deemed to indicate dense scar tissue. Points with voltages between 0.2 and 0.5 mV were defined as indicating damaged but viable tissue. The Lumipoint™ map analysis tool, which automatically identifies fragmented potentials and continuous activation, was used in both maps sequentially on each PV component, in order to assess the presence of PV gaps and the change in the antral potentials after PVI.

**FIGURE 1:**
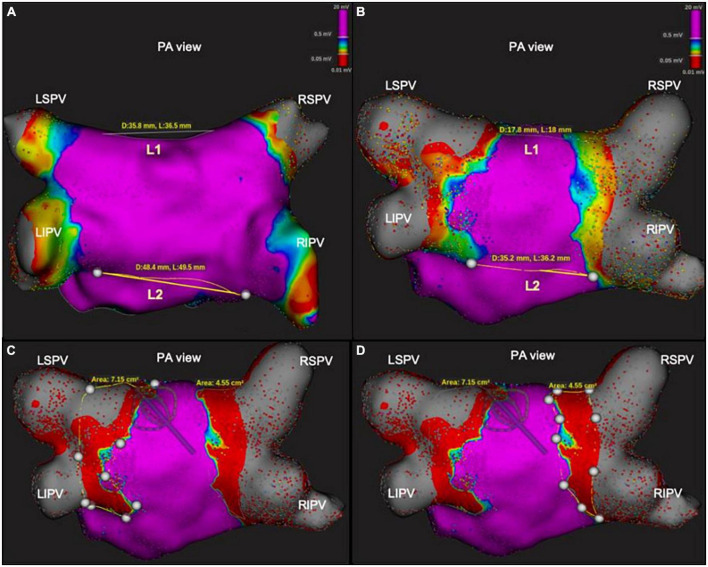
Characterization of cryoablation lesions by measuring distance and area in the posterior wall, before and immediately after ablation, using 3D high-density mapping. All four panels show postero-anterior view of the left atrium of one patient. In the upper panels **(A,B)**, bipolar voltage maps before **(A)** and after **(B)** cryoablation. On the right, the first (L1) and second line (L2) show the posterior distance between the ostium of the superior and inferior PV, respectively; on the left, L1 and L2 show the posterior distance between the two ablated and isolated PV areas. In the lower panels **(C,D)**, examples of measurement of ablated area of the posterior wall with PV-cryoballoon applications.

In our series the PV ostium was identified as the point of maximal inflection between the PV wall and LA wall, and the PV antrum was defined as the region proximal to the PV ostium excluding the PVs whereas the LA posterior wall surface area was defined as the area bordered by the PV lesions and two lines connecting the most superior- and inferior- aspects of the circumferential ablation lines (L1 and L2, respectively), as described in several papers on this topic (17, 18; Figure 1). The surface area of the left and right PV, the ablated antral area of each vein and the non-ablated area of the posterior wall were manually calculated by means of the measurement tool of the Rhythmia™ system. Moreover, another two isthmus lines were manually measured between the right and left anterior carina and the mitral annulus (L3 and L4, respectively) (Supplementary Figure 1).

### Statistical analysis

Descriptive statistics are reported as means ± SD for normally distributed continuous variables, or medians with 25th to 75th percentiles in the case of skewed distribution. Normality of distribution was tested by means of the non-parametric Kolmogorov–Smirnov test. Differences between mean data were compared by means of a *t*-test for Gaussian variables, and the *F*-test was used to check the hypothesis of equality of variance. The Mann–Whitney non-parametric test was used to compare non-Gaussian variables. Differences in proportions were compared by applying χ^2^ analysis or Fisher’s exact test, as appropriate. A *p*-value < 0.05 was considered significant for all tests. All statistical analyses were performed by means of STATISTICA software, version 7.1 (StatSoft, Inc., Tulsa, OK, USA).

## Results

### Study population

Twenty-nine consecutive patients were enrolled. All these patients had a history of paroxysmal AF: duration of the longest AF episode was >1 h in 19 (65.5%) cases; >6 h in 7 (24.1%) cases and >24 h in 3 (10.3%). Besides AF, five (17.2%) patients had a history of atrial flutter/AT. The mean age was 62.1 ± 10 years, and 62% were males (*n* = 18). Twenty-seven patients (93%) experienced mild- to moderate symptoms according to the EHRA AF-related Symptoms Scale. Baseline clinical characteristics are shown in Table 1.

**TABLE 1:** Baseline clinical characteristics of the study population.

Parameter	All patients (*n* = 29)
Age, years	62.1 ± 10
Male gender, n (%)	18 (62.1)
History of AF:	29 (100)
• Duration of the longest episode > 1h:	• 19 (65.5)
• Duration of the longest episode > 6h:	• 7 (24.1)
• Duration of the longest episode > 24h:	• 3 (10.3)
History of atrial flutter/atrial tachycardia:	5 (17.2)
LVEF, %	56 ± 9
Cardiomyopathy, n (%)	6 (20.7)
Hypertension, n (%)	13 (44.8)
Coronary artery disease, n (%)	3 (10.3)
History of heart failure, n (%)	1 (3.4)
COPD, n (%)	1 (3.4)
**EHRA score of AF-related symptoms:**	
• 1—No symptoms	• 1 (3.4)
• 2a—Mild symptoms	• 18 (62.1)
• 2b—Moderate symptoms	• 9 (31.0)
• 3—Severe symptoms	• 1 (3.4)
• 4—Disabling symptoms	• 0 (0.0)

AF, Atrial fibrillation; COPD, Chronic obstructive pulmonary disease; EHRA, European Heart Rhythm Association; LVEF, Left ventricular ejection fraction; PVI, Pulmonary vein isolation.

### Procedural characteristics

A total of 116 PVs were targeted in the whole population and PVI was achieved by means of CB ablation in all but one case, which required an additional radiofrequency touch-up after remapping with the Orion catheter. The mean number of freeze applications per patient was 4.8 ± 1.0 (1.2 ± 0.5 for left inferior PV, 1.2 ± 0.4 for left superior PV, 1.4 ± 0.6 for right inferior PV and 1.1 ± 0.5 for right superior PV). Twelve (41.4%) patients were treated with a single application to each of the PVs. TTI was available in 74 (64.9%) PVs, the median TTI being 38 [30–48] sec (median temperature at TTI = −42°C [−48 to −39]). The median nadir temperature was −56.0°C [−59 to −52], the median time to target at −40°C was 30 [28–34] s, the median thaw time to 0°C was 16 [14–20] s and the median deflation time (from 0 to 20°C) was 23 [20–29] s. Complete occlusion of the PV was achieved in 93 (81.6%) of the PVs treated. Detailed procedural data are reported in Table 2.

**TABLE 2:** Procedural characteristics according to PVs.

Parameter	All PVs	LIPV	LSPV	RIPV	RSPV
Total number of PVs, n	114*	29	29	28	28
Total mapping time, min	14.7 ± 4				
Total ablation time, min	17.9 ± 5				
Total fluoroscopy time, min	18.6 ± 8				
Mean number of CBAs, n	4.8 ± 1.0	1.2 ± 0.5	1.2 ± 0.4	1.4 ± 0.6	1.1 ± 0.5
Single CBA, n (%)	92 (80.7)	24 (82.8)	23 (79.3)	20 (71.4)	25 (89.3)
2 CBAs, n (%)	18 (15.8)	4 (13.8)	6 (20.7)	6 (21.4)	2 (7.1)
>2 CBAs, n (%)	4 (3.5)	1 (3.4)	0 (0.0)	2 (7.2)	1 (3.6)
Patients treated with single CBA of each of the PVs	12 (41.4)				
TTI measured per vein, n (%)	74 (64.9)	19 (65.5)	23 (79.3)	14 (50.0)	18 (64.3)
TTI (s)	38 [30–48]	42 [21–66]	50 [38–70]	43 [33–73]	37 [27–55]
Temperature at TTI (°C)	−42 [−48 to −39]	−47 [−51 to −40]	−52 [−55 to −45]	−48 [−52 to −40]	−49 [−53 to −38]
Time to target (−40°C) (s)	30 [28–34]	30 [28–34]	28 [27–30]	32 [29–35]	29 [27–34]
Nadir temperature (°C)	−56 [−59 to −52]	−54 [−59 to −50]	−58 [−64 to −54]	−55 [−58 to −51]	−57 [−60 to −51]
Thaw time (to 0°C) (s)	16 [14–20]	15 [14–19]	18 [16–21]	15 [12–18]	16 [13–20]
Deflation time (0°C to 20°C) (s)	23 [20–29]	25 [20–30]	25 [20–30]	20 [17–25]	24 [20–25]
Complete occlusion (Occlusion grade = 4)	93 (81.6)	22 (75.9)	27 (93.1)	23 (82.1)	21 (75.0)

PV, Pulmonary Vein; CBA, Cryoballoon application; TTI, Time to isolation; LIPV, Left inferior pulmonary vein; LSPV, Left superior pulmonary vein; RIPV, Right inferior pulmonary vein; RSPV, Right superior pulmonary vein. *A right middle PV was present in two patients, data not included here. “Cardiomyopathy: patients with mild impaired left ventricle ejection fraction (LVEF < 50%); History of heart failure: patients with history of hospitalization for acute heart failure during atrial fibrillation.”

### Assessment of cryothermal lesion extension

**FIGURE 2:**
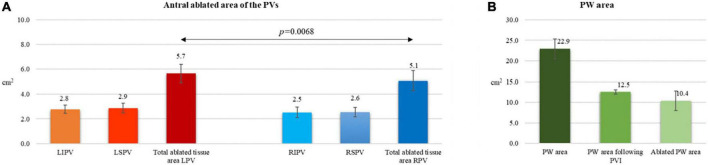
Post-cryoballoon ablation measurements of left atrial ablated areas **(A,B)**. **(A)** Antral ablated area of the PVs. **(B)** Posterior wall area before and following PVI.

**FIGURE 3:**
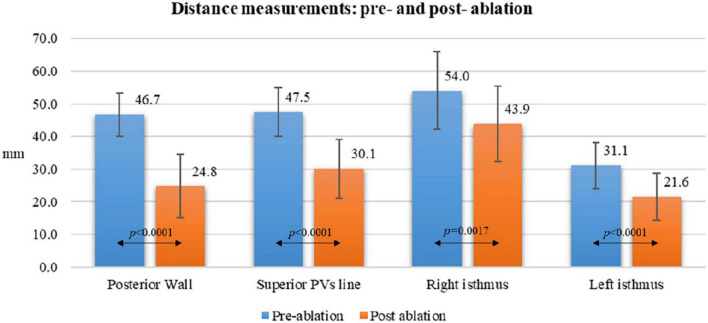
Distance measurements pre- and post-ablation according to different atrial anatomical structures.

Acute lesion characterization was completed in all 29 patients. All the patients were in sinus rhythm at the time of 3D map. Quantitative assessment of the lesions revealed a significant reduction of the antral surface area of the PV, resulting in an ablated area of 5.7 ± 0.7 cm^2^ and 5.1 ± 0.8 cm^2^ for the left PV pair and right PV pair, respectively (*p* = 0.0068). All the lesions were localized in the PV antrum. The mean PW area was 22.9 ± 2 cm^2^ and, following PVI, 44.8 ± 6% (10.4 ± 2 cm2) of the PW area was ablated, as shown in Figures 2A,B. In five (17%) cases, the ablated area was larger than 50% of the initial PW area. The distance measurements between lesions created in the PW (L1), in the superior PV line (L2) and at the right (L3) and left (L4) isthmus showed a significant reduction after PVI (L1: 46.7 ± 7 mm vs. 24.8 ± 10 mm, *p* < 0.0001, 46.4 ± 20% reduction; L2: 47.5 ± 7 mm vs. 30.1 ± 9 mm, *p* < 0.0001, 36.1 ± 18% reduction; L3: 54.0 ± 12 mm vs. 43.9 ± 11 mm, *p* = 0.0017, 18.6 ± 11% reduction; L4: 31.1 ± 7 mm vs. 21.6 ± 7 mm, *p* < 0.0001, 27.6 ± 24% reduction). Details are reported in Figure 3. Figure 1 and Supplementary material show an example of the pre- and post-ablation high-definition maps used to quantify the extent of ablation on the left atrium PW and the distances between lesions.

### Characteristics of low-voltage potentials within the antral region, on confirmatory high-density mapping

**FIGURE 4:**
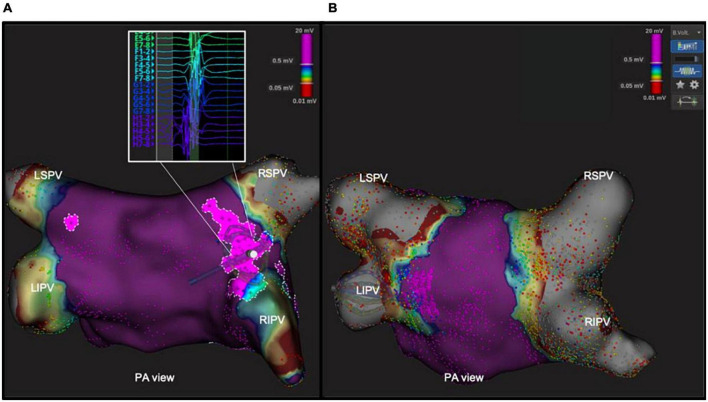
Abnormal area of fragmented potentials within the antral area, revealed by Lumipoint™ tool before and after ablation. In the left panel **(A)**, postero-anterior view of the left atrium with the Lumipoint™ tool activated, showing an area of fragmented potential around the antrum of right PVs and on the carina between the veins, and a small area around the LSPV. The Orion high-density mapping catheter in that area shows fragmented signals with more than seven deflections on the *splines G* and *H.* In the right panel **(B)**, the same tool was turned on after the ablation, and showed the absence of the previously identified fragmented signals within the antrum.

Before PVI, fragmented potentials (mean 205 area of 2.7 ± 0.7 cm2) were detected by means of the Lumipoint™ tool between the antral region of the PV and the PW [11.7 ± 3.4% of the entire PW area in 11 (38%) patients]. The most frequent location of these signals was around the posterior carina of the right and left PVs, the anterior carina of the right PV and the left posterior roof. After PVI, all these areas proved to have been included in the antral ablation circles, and the Lumipoint™ tool revealed no fragmented potentials around the PV. An example of these potentials before and after the procedure is shown in Figure 4.

### Outcome

**FIGURE 5:**
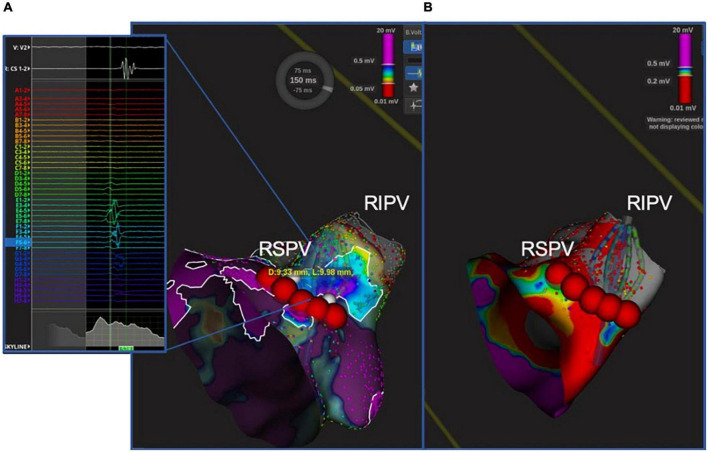
Unidentified PV potential in the right inferior PV after cryoablation; re-isolation was necessary. In one patient, the Orion™ high-density mapping catheter was able to identify PV potentials after ablation, despite the absence of PV potentials on decapolar circular mapping (POLARMAP). In the right panel **(B)**, the electroanatomical map and activation map analyzed by means of Lumipoint™ show a 9.98 mm gap in the posteroinferior portion of the RIPV and the presence of low-amplitude fragmented signals in that region, as shown on *splines E, F*, and *G.* Five RF applications were able to isolate the vein and eliminate the previously recorded signals [Left panel **(A)**].

After CB ablation, complete isolation of each PV was documented by the POLARMap™ catheter in all patients. By contrast, confirmatory high-density mapping through the Orion™ catheter and the Lumipoint™ tool unveiled PV signals in 1 out of 114 of the PVs (0.9%–1 patient with PV gap: 3.5%). These signals were associated with the presence of a visual gap on the LA bipolar UHDM and activation map (linear extension of 9.98 mm with a mean voltage of 0.45 mV at the gap spot) located in the postero-inferior portion of the right inferior PV (Figure 5). Five touch-up RF applications at 35W were performed to completely isolate the PV, with a mean local impedance drop of 15 ohms. At the end of the procedure, all PVs were checked again with the high-density catheter, and proved to have been successfully isolated. Over 30-day follow-up, no major procedure-related adverse events were reported. After a mean follow-up of 333 ± 74 days, 3 patients (10.3%) suffered an AF/AT recurrence after 90-day blanking period. All recurrences reordered were AF; after the index procedure, time to recurrence, respectively for the three patients, was 6, 8, and 9 months after the index procedure. Ten patients (34%) were on antiarrhythmic treatment at the first follow-up visit.

## Discussion

To the best of our knowledge, this is the largest study to evaluate the acute lesion extension, the effect on the antral fragmented electrogram and the rate of unidentified PV signals after CB ablation by means of a novel system (POLARx™ Cryoablation) using left atrium 3D UHDM in patients with paroxysmal AF. The main findings of our study are: (1) the lesions created with the new CB ablation system involve the PV antrum, with about 50% of the PW remaining untouched; (2) the new system, which uses a short-tip CB and a circular mapping catheter, failed to achieve PVI in only 0.8% of all PVs treated; (3) antral fragmented potentials were completely eliminated by CB ablation, without any residual antral potentials being identified by Lumipoint™; (4) the novel CB system is a safe and effective means of achieving PV occlusion and isolation.

### Extension of acute ablation lesions created by the POLARx™ cryoballoon system, as assessed with the basket catheter

The atrial antrum seems to be important in the physiopathology of atrial fibrillation, and PVI by means of a wide antral approach is reported to yield better results than ostial PVI in terms of freedom from total atrial tachyarrhythmia recurrence during long-term follow-up (19). In our observational multicenter study, all the PV lesions were antral, the ablated antral area being 5.7 cm^2^ and 5.1 cm^2^ in left and right PVs, respectively; on average, 45% of the PW was ablated, and more than 50% was ablated in 17% of patients. In the right septal antrum and on the left anterior ridge, the maps also showed an effective reduction of the isthmus line. These data are similar to those from previous studies using the Arctic Front cryoballon system (6, 9). A recent study analyzed the ablation lesion set in a subpopulation of nine patients who had undergone cryoablation by means of the POLARx™ system with the aid of a circular mapping catheter (10). The authors reported that 50% (44–59%) of the posterior wall had been ablated by the CB, with no difference in the antral surface area of ablation between right and left PVs. In our study, the use of a basket catheter with 64 small electrodes yielded the same results in terms of ablation extension; moreover, it allowed us to qualitatively assess the presence of notch-like normal voltage areas on the left posterior side of the carina, which had not been possible in the previous study (Figure 1).

### Role of high-resolution basket mapping catheter in identifying pulmonary vein gaps

The conventional circular mapping catheter used in CB ablation to assess the PVs may fail to detect small strains of conductive tissue in the PV, leading to an increased rate of arrhythmia recurrence due to incomplete PVI during the index ablation procedure. This shortcoming of the conventional mapping catheter is due to the large electrode size, the large inter-electrode distance and the oblique orientation of the catheter once it is positioned inside the PV. In this setting, the 64-electrode basket UHDM catheter is superior in detecting PV potentials after ablation, especially around the antrum-ostium of the PV, where its greater stability enables it to acquire points more effectively. In previous studies, an 8-pole circular catheter used in combination with a short-tip balloon was compared with a basket catheter (Orion™ catheter). The 8-pole catheter missed PV potentials in 24% of cases in which an older-generation balloon was used and in 1.4% of procedures done with a fourth-generation balloon (11, 12). In our study, the discordance between the new POLARx™ circular catheter used in combination with a short-tip balloon and the Orion basket catheter was only 0.9%. The one case in which a PV gap was detected, it was located on the postero-inferior tract of the right inferior PV; the gap was wide and had a mean Voltage of 0.45 mV and was successfully and easily ablated by means of radiofrequency touch-up applications. This finding is in line with that of a multicenter study that analyzed PV gaps by means of an UHDM basket catheter on redo procedures; after cryoablation, gaps are fewer but wider, and voltage mapping seems to be better able to detect them than during post-RF procedures (20).

### Elimination of antral fragmented potentials identified by the Lumipoint™ tool

Fractionated signals in atrial muscle tissue cause repetitive atrial firing; this increases susceptibility to AF and has been used as an indicator of atrial vulnerability. A recent study aimed at identifying the best number of peak slider deflections that correlated with non-PV triggers in the left atrium suggested that, on using the Lumipoint™ tool to identify fragmented potentials (>7 deflections) could cause repetitive atrial firing (21). Moreover, in a recent study, local residual antral potentials were easily discernible by means of the UHDM system and Lumipoint™ software, in both *de novo* and redo patients, when no PV conduction was present. In our population, CB ablation was able to create bilateral wide antral lesions that included all areas of fragmented electrograms identified by the Lumipoint™ tool in patients with paroxysmal AF, without additional PV applications. The same tool, when used to check for the presence of residual antral electrograms, highlighted the absence of previously recorded abnormal signals after ablation.

### Limitations

The present study has some limitations. First, the study group was small (only 29 patients) and there was no control group undergoing RF ablation. Although the results are concordant with those of a previous study, a larger study is needed in order to assess the discordance between the POLARMap™ catheter and the basket catheter in detecting unidentified PV potentials and to check for the elimination of all antral potentials assessed by means of Lumipoint™. Second, this was a multicenter study involving four Italian centers with inter-center variability; however, there is less inter-operator variability in CB ablation procedures. “Finally, the significant variability of PV-LA anatomy may have impacted the quantitatively assessment of the antral region or the posterior wall region and derived parameters on the ablation effects.”

## Conclusion

Pulmonary vein isolation by means of this novel cryoballoon created wide antral lesions and eliminated antral fragmented potentials. The new system, with short tip and circular mapping catheter, failed to achieve PV isolation in only 0.9% of all PVs treated.

## Data availability statement

The original contributions presented in this study are included in the article/Supplementary material, further inquiries can be directed to the corresponding author.

## Ethics statement

The studies involving human participants were reviewed and approved by Catholic University of Sacred Heart, Rome, Italy. The patients/participants provided their written informed consent to participate in this study.

## Author contributions

FS, MN, and GPe: concept, design, data analysis, interpretation, and drafting manuscript. GB, FPe, AB, GPi, RM, FT, AF, and AD: data analysis, interpretation, and data collection. CT, GS, SI, and FC: drafting manuscript. MM and FPi: interpretation of the data and critical revision of manuscript. All authors contributed to the article and approved the submitted version.
