# Supplementary material for: Ultra-high-resolution assessment of lesion extension after cryoballoon ablation for pulmonary vein isolation
Source: Front Cardiovasc Med. 2022 Nov 9;9:985182. doi: 10.3389/fcvm.2022.985182 (PMC9681817; doi:10.3389/fcvm.2022.985182)

Supplemental material Fig. 1. Characterization of cryoablation lesions on the left and right isthmus, before and immediately after ablation, using 3D high-density mapping

In the **Upper panels**, bipolar voltage maps in left-lateral view before (on the left) and after (on the right) cryoablation. The line L4 measures the distance between the left carina ridge and the mitral annulus on the right, and between the ablated area and the mitral annulus on the left.

In the **Lower panels**, bipolar voltage maps in right-lateral view before (on the left) and after (on the right) cryoablation. The line L3 measures the distance between the right anterior carina and the mitral annulus on the right, and between the ablated area and the mitral annulus on the left.

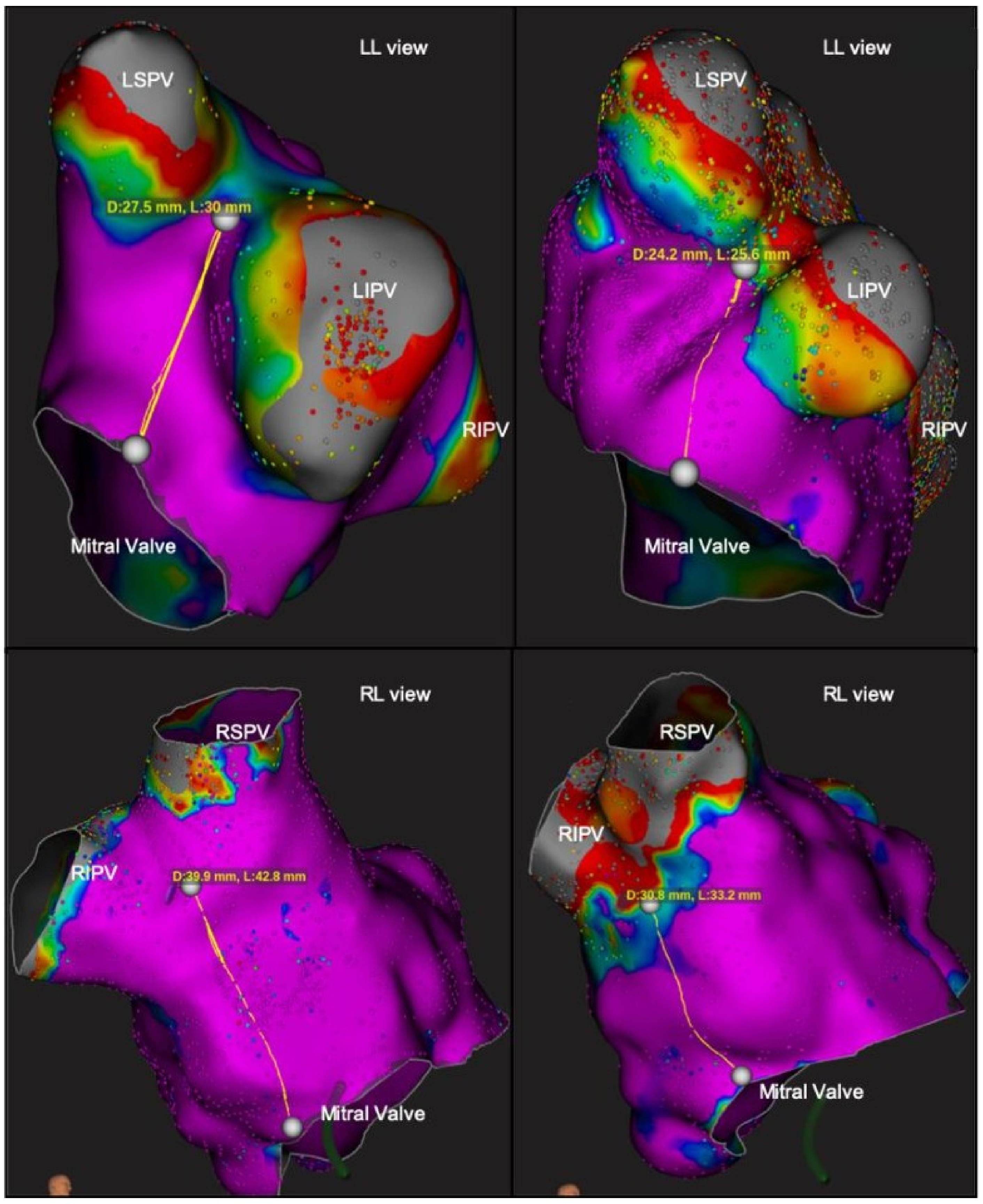

Supplement: Supplementary file 1 [file Image_1.pdf]
